# Artemisinin Confers Cytoprotection toward Hydrogen Peroxide-Induced Cell Apoptosis in Retinal Pigment Epithelial Cells in Correlation with the Increased Acetylation of Histone H4 at Lysine 8

**DOI:** 10.3390/molecules29081789

**Published:** 2024-04-15

**Authors:** Chao Yang, Lijun Ge, Xiyong Yu, Philip Lazarovici, Wenhua Zheng

**Affiliations:** 1 Department of Pharmaceutical Science, Faculty of Health Sciences, University of Macau, Taipa 999078, Macau, China; yb97634@um.edu.mo (C.Y.); gelijun@zcmu.edu.cn.com (L.G.); 2 College of Life Science, Zhejiang Chinese Medical University, Hangzhou 310053, China; 3 School of Pharmaceutical Sciences, Guangzhou Medical University, Guangzhou 511436, China; yuxycn@gzhmu.edu.cn; 4 School of Pharmacy Institute for Drug Research, Faculty of Medicine, The Hebrew University of Jerusalem, Jerusalem 9112002, Israel; philipl@ekmd.huji.ac.il; 5 Zhuhai UM Science & Technology Research Institute, Zhuhai 519000, China

**Keywords:** Artemisinin, H_2_O_2_, apoptosis, Acetyl-H4 (Lys 8), retinal pigment epithelial cells, cytoprotection

## Abstract

Increased oxidative stress is one of the critical pathologies inducing age-related macular degeneration (AMD), characterized by retinal pigment epithelial (RPE) cell damage and death. The unbalanced acetylation and deacetylation of histones have been implicated in AMD pathogenesis or hydrogen peroxide (H_2_O_2_)-induced cell damage. Therefore, strategies aimed at controlling the balance between acetylation and deacetylation may effectively protect RPE cells from oxidative damage. Artemisinin is an antimalarial lactone drug derived from *Artemisia annua*, with antioxidant activity known to modulate histone acetylation in the brain, but its effect on the retina is unknown. In this study, we aimed to investigate whether Artemisinin exerts a cytoprotective effect on oxidative stress-induced apoptosis in RPE cells by regulating histone acetylation. We hypothesized that Artemisinin confers cytoprotection toward H_2_O_2_-induced apoptosis in RPE cells through this mechanism. In the present study, we found that Artemisinin at a sub-clinic dosage of 20 μM inhibited the H_2_O_2_-induced cell viability decrease and B-cell lymphoma 2 (Bcl-2) protein level decrease and attenuated the H_2_O_2_-induced decrease in the histone H4 lysine (Lys) 8 acetylation [Acetyl-H4 (Lys 8)] level in the retinal RPE cell line D407. As expected, histone deacetylase inhibitor Trichostatin A at the concentration of 250 nM increased the Acetyl-H4 (Lys 8) level in D407 cells and attenuated the H_2_O_2_-induced cell viability decrease and apoptosis. Similar findings were obtained using adult RPE (ARPE)19 cells, another human RPE cell line, and primary human RPE cell cultures. In conclusion, these results confirmed our hypothesis and indicated that Artemisinin attenuated H_2_O_2_-induced apoptosis in apparent correlation with the increase in the Acetyl-H4 (Lys 8) level, which is associated with gene transcription and cell survival. By modulating histone acetylation, Artemisinin may restore the balance between acetylation and deacetylation and enhance the resistance and survival of RPE cells under oxidative stress. Our study provides novel mechanistic insights into the effect of Artemisinin on histone acetylation and apoptosis in RPE cells and supports the potential application of Artemisinin in the prevention and/or treatment of AMD.

## 1. Introduction

Age-related macular degeneration (AMD) is a leading cause of vision loss in the elderly [1]. Oxidative stress-induced retinal pigment epithelial (RPE) cell damage is considered the initial trigger of AMD [2]. Oxidative stress refers to the imbalance between the production and clearance of reactive oxygen species (ROS) in cells, resulting in cellular structure and function damage [3]. ROS can cause oxidative damage to RPE cells through various pathways, including lipid peroxidation, protein oxidation, DNA damage, mitochondrial dysfunction, apoptosis, and autophagy [3]. These damages affect the normal physiological functions of RPE cells, such as phagocytosis of outer retinal discs, secretion of growth factors, maintenance of retinal vascular barrier, etc. [4]. The damage and dysfunction of RPE cells further lead to the degeneration and death of retinal photoreceptor cells, and/or choroidal neovascularization, resulting in central vision loss [4]. Therefore, oxidative stress plays a key role in the pathogenesis of AMD. It was reported that a critical epigenetic change, histone modification, is involved in retinal diseases such as AMD [5,6]. The chromatin is characterized by a stable interaction of DNA with histones and non-histone proteins. Histones help in compressing the nuclear DNA to form nucleosome-macromolecular structures with different histones such as H2A, H2B, H3, and H4 [7]. Histones are involved in the regulation of DNA transcription, replication, repair, and chromatin packing through several different post-translational modifications [8]. One of the post-translational modifications of histones is acetylation by histone acetyltransferases (HATs), a class of enzymes that catalyze the transfer of acetyl groups to conserved lysine residues at the tail of histones, facilitating the relaxation of chromatin that is transcriptionally active [9]. Conversely, histone deacetylases (HDACs) catalyze the removal of acetyl groups from histones, resulting in more tightly packed chromatin that is transcriptionally inactive [9]. Previous studies reported that the acetylation of lysine (Lys) residues of histone H3 (Lys 9 and Lys 14) or H4 (Lys 8) N-terminal tail was observed in healthy retinal rod photoreceptors of eyes [10,11], and decreased levels of histone H4 acetylation were found in apoptotic retinal ganglion cells [12].

Research reported that natural products were highly potential therapeutic agents for the treatment of AMD, based on their mechanisms of action: anti-oxidative stress, anti-inflammation, and anti-neovascularization [13]. Artemisinin is a natural compound that has been extensively used in clinical practice as an antimalarial drug. Artemisinin can ameliorate oxidative damage in retinal pigment epithelial cells and neuronal cells [14,15,16]. Artemisinin enhanced histone H3 and H4 acetylation levels in brain tissues [17], but its effects on histone H3 and H4 in the retina are unknown. Both D407 and adult RPE (ARPE)19 are human RPE cell lines that are widely used for the AMD model. Therefore, both D407 and ARPE19 cells were used to test the effect of Artemisinin, which can increase the validity and generalizability of our findings. Exposure to hydrogen peroxide (H_2_O_2_) is a widely used method for inducing oxidative stress in cell culture models [18]. In this study, we investigated the acetylation levels of histone H3 at Lys 9 and histone H4 at Lys 8 in RPE cells, under exposure to H_2_O_2_ in RPE cell cultures, as well as the effect of Artemisinin on these histone acetylation levels. We propose that the cytoprotective effect of Artemisinin toward H_2_O_2_-induced apoptosis in RPE cells is correlated to the histone acetylation.

## 2. Results

### 2.1. H_2_O_2_ Decreased Cell Viability, Increased Cytotoxicity, and Caused Reactive Oxygen Species (ROS) Generation in D407 Cell Cultures

**Figure 1:**
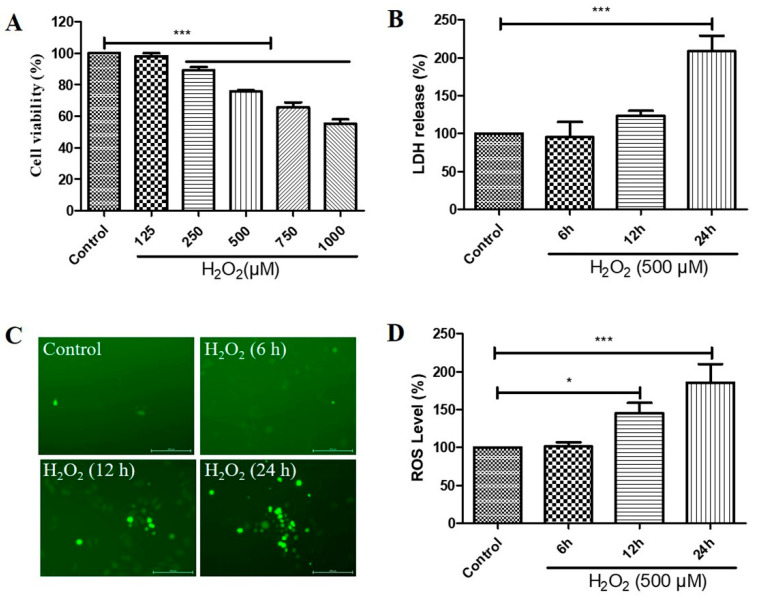
Hydrogen peroxide (H_2_O_2_)-induced a decrease in cell viability in a concentration-dependent manner and increases in cytotoxicity and reactive oxygen species (ROS) levels in a time-dependent manner, in D407 cell cultures. (**A**) D407 cells attached to the 96-well plate were treated with different concentrations of H_2_O_2_ (125–1000 μM) for 24 h. Cell viability was detected using 3-(4,5-dimethylthiazol-2-yl)-2,5-diphenyltetrazolium bromide (MTT). (**B**) The cell cultures were treated with 500 μM H_2_O_2_ for several time points (6–24 h). Cytotoxicity was measured by lactate dehydrogenase (LDH) release. (**C**,**D**) The cell cultures were treated with 500 μM H_2_O_2_ for several time points (6–24 h). ROS levels were detected using the DCFH-DA probe. The scale bar is 200 μm. Untreated cells were used as control. * indicates *p* < 0.05, *** indicates *p* < 0.001.

We first investigated the effects of different concentrations of H_2_O_2_ treatment on the cell viability and different durations of H_2_O_2_ treatment on the cytotoxicity and ROS levels of D407 cell cultures. We found that H_2_O_2_ treatment from 250 to 1000 μM caused a significant decrease in cell viability compared with the control untreated group, as measured by the 3-(4,5-dimethylthiazol-2-yl)-2,5-diphenyltetrazolium bromide (MTT) assay (Figure 1A). Incubation for 24 h with 500 μM H_2_O_2_ caused significant cytotoxicity, as evidenced by the increased lactate dehydrogenase (LDH) release (Figure 1B). Furthermore, we observed that increasing the incubation time with 500 μM H_2_O_2_ for 6 h, 12 h, and 24 h caused a gradual increase in ROS levels, as detected by the DCFH-DA fluorescence probe (Figure 1C,D). These findings suggest that H_2_O_2_ treatment induces oxidative stress and impairs the function and survival of D407 cells.

### 2.2. H_2_O_2_ Decreased Histone H4 (Lys 8) and Histone H3 (Lys 9) Acetylation Levels in D407 Cell Cultures

**Figure 2:**
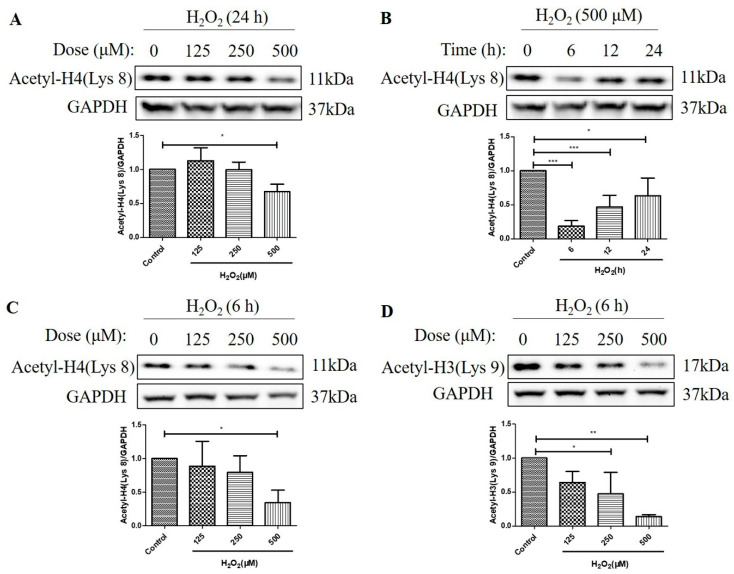
H_2_O_2_ decreased acetylation levels of the histone H4 (Lys 8) and the H3 (Lys 9) in D407 cell cultures. (**A**) The cells were treated with different concentrations of H_2_O_2_ (125, 250, or 500 μM) for 24 h. (**B**) The cells were treated with 500 μM H_2_O_2_ for several time points (0–24 h). (**C**,**D**) The cells were treated with different concentrations of H_2_O_2_ (125, 250, or 500 μM) for 6 h. Untreated cells were used as the control. The histone H4 (Lys 8) acetylation [Acetyl-H4 (Lys 8)] and histone H3 (Lys 9) acetylation [Acetyl-H3 (Lys 9)] levels and glyceraldehyde-3-phosphate dehydrogenase (GAPDH) protein level for normalization were detected by western blotting and Image J (version 1.51d) quantified protein bands. * indicates *p* < 0.05, ** indicates *p* < 0.01, *** indicates *p* < 0.001.

We then investigated the effects of H_2_O_2_ treatment on the acetylation levels of histone H4 (Lys 8) and histone H3 (Lys 9) in D407 cell cultures. We found that H_2_O_2_ treatment for 24 h caused about a 33% decrease in acetylation level of histone H4 (Lys 8) at 500 μM H_2_O_2_ compared with the control group, as measured by western blotting (Figure 2A). Moreover, 500 μM H_2_O_2_ reduced the acetylation level of histone H4 (Lys 8) more significantly, at 6 h (84%) than at 12 h (52%) and 24 h (37%) compared with the control group (Figure 2B). Furthermore, we observed that H_2_O_2_ treatment (125 μM, 250 μM, or 500 μM) for 6 h caused a dose-dependent decrease in acetylation levels of both histone H4 (Lys 8) and histone H3 (Lys 9) as compared with the control group (Figure 2C,D). The maximal concentration of 500 μM caused 65 and 86% strong decreases in the acetylation levels of histone H4 (Lys 8) and histone H3 (Lys 9), respectively. These findings indicate that H_2_O_2_ treatment affects the acetylation status of different histone residues in a dose-dependent manner and that histone H3 (Lys 9) is more sensitive to H_2_O_2_-induced deacetylation than histone H4 (Lys 8).

### 2.3. HDAC Protein Levels Were Not Upregulated after the Treatment with H_2_O_2_ for 6 h in D407 Cell Cultures

**Figure 3:**
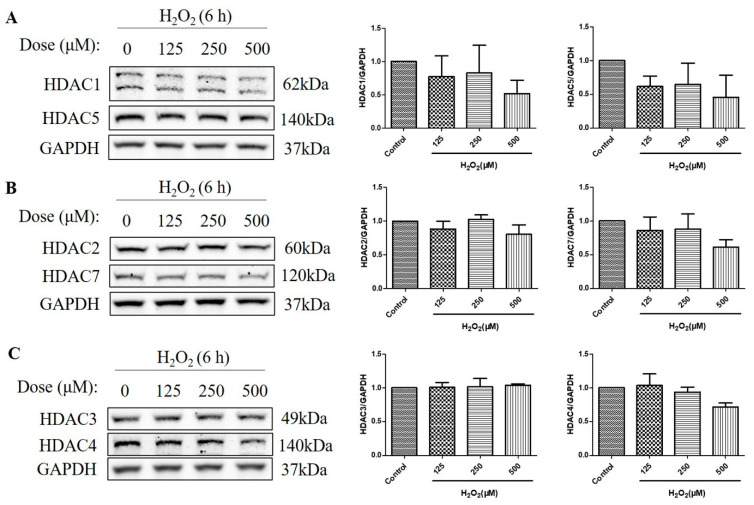
H_2_O_2_ did not increase class I/IIα histone deacetylases (HDACs) protein levels in D407 cell cultures at 6 h. (**A**–**C**) The cells were treated with different concentrations of H_2_O_2_ (125, 250, or 500 μM) for 6 h. Cells not exposed to H_2_O_2_ were used as the control. The protein levels of class I HDACs (HDAC1, HDAC2, and HDAC3) and class IIα HDACs (HDAC4, HDAC5, and HDAC7) were detected by western blotting and protein bands were quantified by Image J.

HDACs are classified into four classes based on their sequence homology and domain organization: Class I (HDACs 1, 2, 3, and 8), Class II (HDACs 4, 5, 6, 7, 9, and 10), Class III (Sirtuins 1–7), and Class IV (HDAC11) [19]. As HDACs can deacetylate histone H3 and H4 [20], and the over-activation of HDAC is detrimental to retinal health [21,22], we then measured the protein level of a series of HDACs, including class I HDACs (HDAC1, HDAC2, and HDAC3) and class IIα HDACs (HDAC4, HDAC5, and HDAC7). The HDAC protein levels were measured at 6 h, as H_2_O_2_-reduced histone acetylation levels were more significant at this time. We found that H_2_O_2_ treatment for 6 h did not increase the HDAC1, HDAC2, HDAC3, HDAC4, HDAC5, and HDAC7 protein levels, as measured by western blotting (Figure 3A–C). These results suggest that the decreased acetylation of the histone H4 (Lys 8) and the histone H3 (Lys 9) is not regulated by class I and class IIα HDACs but by other mechanisms, such as the activation of Class III HDACs (Sirtuins) or the inhibition of HATs.

### 2.4. Artemisinin Corrected H_2_O_2_-Induced Cell Viability Decline, and Decrease in Histone H4 (Lys 8) Acetylation Level in D407 Cell Cultures

**Figure 4:**
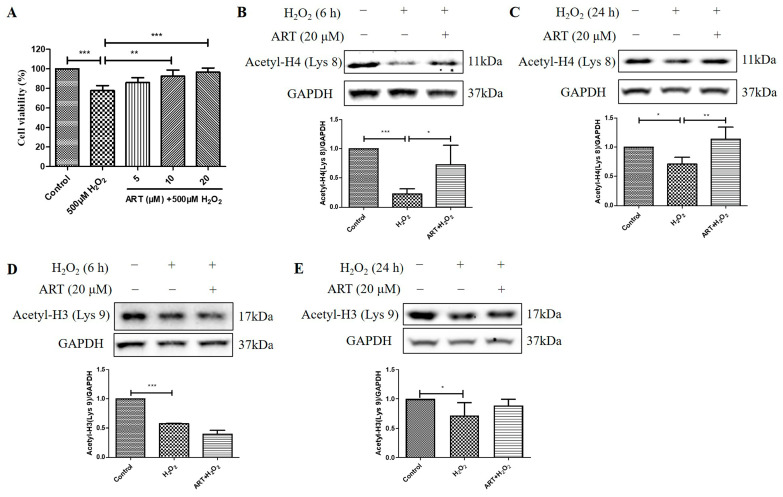
Artemisinin corrected H_2_O_2_-induced inhibition of cell viability and histone H4 (Lys 8) acetylation level in D407 cell cultures. (**A**) The cells were treated with or without 20 μM Artemisinin (ART) for 1 h, followed by incubation with 500 μM H_2_O_2_ for 24 h. Cell viability was detected using MTT. (**B**–**E**) The cells were treated with or without 20 μM Artemisinin for 1 h, followed by incubation with 500 μM H_2_O_2_ for 6 h or 24 h. Acetyl-H4 (Lys 8) and Acetyl-H3 (Lys 9) levels, and GAPDH protein level for normalization, were detected by western blotting, and Image J quantified protein bands. * indicates *p* < 0.05, ** indicates *p* < 0.01, *** indicates *p* < 0.001.

We then investigated the effects of Artemisinin pretreatment on the viability and acetylation levels of histone H4 (Lys 8) and histone H3 (Lys 9) in D407 cell cultures exposed to H_2_O_2_. We found that Artemisinin pretreatment for 1 h conferred a dose-dependent manner protection toward the H_2_O_2_-induced cell viability decline in D407 cell cultures, as measured by the MTT assay (Figure 4A). In parallel experiments, Artemisinin pretreatment for 1 h significantly suppressed the H_2_O_2_-induced reduction in histone H4 (Lys 8) acetylation [Acetyl-H4 (Lys 8)] level at 6 h and 24 h, as detected by western blotting (Figure 4B,C). In contrast, Artemisinin pretreatment for 1 h did not suppress H_2_O_2_-induced reduction in histone H3 (Lys 9) acetylation [Acetyl-H3 (Lys 9)] level at 6 h and 24 h significantly (Figure 4D,E), indicative of a selective effect on Acetyl-H4 (Lys 8). These results suggest that Artemisinin protects D407 cells from H_2_O_2_-induced oxidative injury by modulating histone acetylation, especially histone H4 (Lys 8), which may be involved in the regulation of genes related to oxidative stress response and survival.

### 2.5. Artemisinin Reversed H_2_O_2_-Induced Downregulation of B-Cell Lymphoma 2 (Bcl-2) to Bcl-2 Associated X-Protein (Bax) Protein Ratio in D407 Cell Cultures

**Figure 5:**
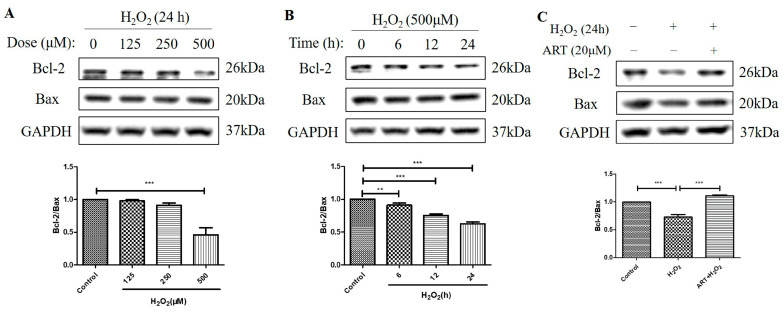
Artemisinin abolished the H_2_O_2_-induced decrease in the B-cell lymphoma 2 (Bcl-2) to Bcl-2 Associated X-protein (Bax) protein ratio in D407 cell cultures. (**A**,**B**) The cells were treated with different concentrations of H_2_O_2_ for 24 h, and 500 μM H_2_O_2_ for different time points (0–24 h). (**C**) The cells were pretreated with or without 20 μM Artemisinin for 1 h, followed by incubation with 500 μM H_2_O_2_ for 24 h. Untreated cells were used as the control. Protein levels of Bcl-2, Bax, and GAPDH for normalization were detected by western blotting, and Image J quantified protein bands. ** indicates *p* < 0.01, *** indicates *p* < 0.001.

We further investigated the effects of H_2_O_2_ treatment and Artemisinin pretreatment on the protein levels of Bcl-2 and Bax and the ratio of Bcl-2 to Bax in D407 cell cultures. Bcl-2 is an anti-apoptotic protein, while Bax is a pro-apoptotic protein in the mitochondrial apoptosis pathway [23]. The ratio of Bcl-2 to Bax determines the susceptibility of cells to apoptotic stimuli, such as oxidative stress. We found that H_2_O_2_-induced oxidative stress resulted in decreased Bcl-2 protein level whereas the protein level of Bax was unchanged, suggesting that the regulation of Bcl-2, but not of Bax, contributed to H_2_O_2_-induced apoptosis in D407 cell cultures. Protein quantification results showed that H_2_O_2_ significantly decreased the protein ratio of Bcl-2 to Bax (approximately by 40~50%, at 500 μM for 24 h) in a dose- and time-dependent manner (Figure 5A,B). This indicates that H_2_O_2_ treatment shifts the balance of Bcl-2 and Bax toward apoptosis. Artemisinin pretreatment significantly reversed the H_2_O_2_-induced decrease in the Bcl-2 to Bax protein ratio at 24 h (Figure 5C), as expected from its cytoprotective effect. This suggests that Artemisinin pretreatment restores the balance of Bcl-2 and Bax and prevents H_2_O_2_-induced mitochondrial apoptosis in D407 cell cultures.

### 2.6. Trichostatin A (TSA) Reversed H_2_O_2_-Induced Cell Viability Decline, Apoptosis, and Decrease in Histone H4 (Lys 8) Acetylation Levels in D407 Cell Cultures

**Figure 6:**
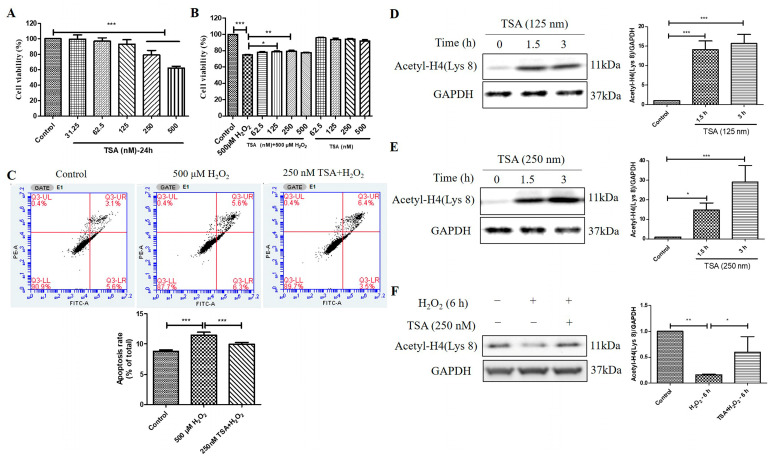
Trichostatin A (TSA) protected D407 cell cultures from H_2_O_2_-induced cell viability decline, and apoptosis, and increased the acetylation level of histone H4 (Lys 8) in D407 cell cultures. (**A**) The cells were treated with different concentrations of TSA (31.25–500 μM) for 24 h. Cell viability was measured using MTT. (**B**) The cells were treated with or without TSA for 2 h, followed by incubation with or without H_2_O_2_ (500 μM) for 24 h. Cell viability was measured using MTT. (**C**) The cells were pretreated with or without TSA (250 nM) for 2 h, followed by incubation with H_2_O_2_ (500 μM) for 24 h. Cell apoptosis was detected by flow cytometry using Annexin V and PI staining. (**D**,**E**) The cells were treated with TSA (125 nM or 250 nM) for 1.5 h or 3 h. Untreated cells were used as the control. (**F**) The cells were treated with or without TSA (250 nM) for 1 h, followed by incubation with H_2_O_2_ (500 μM) for 6 h. Acetyl-H4 (Lys 8) level and GAPDH protein level were detected by western blotting, and Image J quantified protein bands. * indicates *p* < 0.05, ** indicates *p* < 0.01, *** indicates *p* < 0.001.

TSA is a potent and specific inhibitor of HDACs. We found that TSA treatment (250 nM and 500 nM) for 24 h had an inhibitory effect on cell viability in D407 cell cultures, as measured by the MTT assay (Figure 6A). However, pretreatment with 125 nM and 250 nM TSA for 2 h protected the cell cultures from cell viability decline caused by H_2_O_2_ (Figure 6B). This indicates that TSA pretreatment confers cytoprotection against H_2_O_2_-induced oxidative injury. Moreover, 250 nM TSA pretreatment inhibited H_2_O_2_-induced cell apoptosis, as evidenced by flow cytometry using Annexin V-FITC and propidium iodide (PI) staining (Figure 6C). The increased Acetyl-H4 (Lys 8) level after the acute treatment of 125 nM and 250 nM TSA was identified by western blotting analysis (Figure 6D,E). Furthermore, the TSA pretreatment for 1 h significantly suppressed H_2_O_2_ -induced reduction in Acetyl-H4 (Lys 8) level at 6 h (Figure 6F). These results suggest that the short-term elevated acetylation by TSA could protect cells from H_2_O_2_-induced damage by modulating cell viability, apoptosis, and histone acetylation in D407 cell cultures.

### 2.7. The Cytoprotective Effect of Artemisinin toward H_2_O_2_-Induced Apoptosis in ARPE19 Cell Cultures Is Correlated to Reversal of the Histone H4 (Lys 8) Acetylation Level Decline

**Figure 7:**
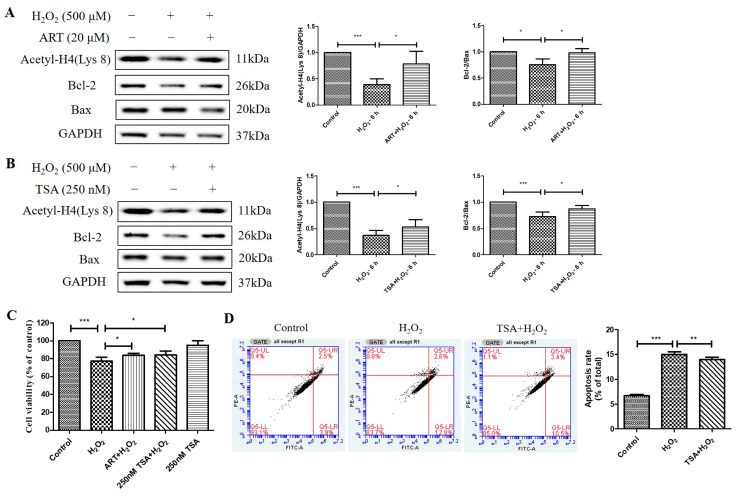
Artemisinin and TSA conferred cytoprotection toward H_2_O_2_-induced cell apoptosis in adult RPE (ARPE)19 and primary human RPE cells. (**A**,**B**) ARPE19 cells attached to the 24-well plate were treated with or without 20 μM Artemisinin or 250 nM TSA for 1 h, followed by incubation with 500 μM H_2_O_2_ for 6 h. Acetyl-H4 (Lys 8) level, and Bcl-2, Bax, and GAPDH protein levels were detected by western blotting, and Image J quantified protein bands. (**C**) Primary human RPE cell cultures were pretreated with or without 20 μM Artemisinin or 250 nM TSA for 1 h, and then treated with or without 500 μM H_2_O_2_ for 24 h. Cell viability was detected using MTT. (**D**) Primary human RPE cells were attached to the 12-well plate and treated with or without 250 nM TSA for 1 h, followed by incubation with 500 μM H_2_O_2_ for 24 h. Cell apoptosis was detected by flow cytometry using Annexin V and PI staining. * indicates *p* < 0.05, ** indicates *p* < 0.01, *** indicates *p* < 0.001.

At last, we investigated the effects of Artemisinin and TSA pretreatment on the acetylation level of histone H4 (Lys 8) and the ratio of Bcl-2 to Bax in ARPE19 cells exposed to H_2_O_2_. We found that Artemisinin or TSA pretreatment for 1 h significantly suppressed the H_2_O_2_-induced reduction in Acetyl-H4 (Lys 8) level and Bcl-2 to Bax protein ratio at 6 h in ARPE19 cells, as indicated by the western blotting results. (Figure 7A,B). Furthermore, the MTT assay results confirmed that pretreatment with 20 μM Artemisinin or 250 nm TSA for 1 h conferred significant cytoprotection to primary human RPE cell cultures from cell viability decrease induced by H_2_O_2_ insult (Figure 7C). Pretreatment of the cultures with 250 nm TSA also inhibited H_2_O_2_-induced apoptosis in primary human RPE cells, as evidenced by flow cytometry using Annexin V and PI staining (Figure 7D). These results suggest that Artemisinin and TSA pretreatment increase histone acetylation and modulate the balance of Bcl-2 and Bax, thereby protecting ARPE19 and primary human RPE cells from H_2_O_2_-induced oxidative injury and apoptosis.

## 3. Discussion

H_2_O_2_ is a kind of ROS donor that can cause oxidative stress and damage to cells, leading to apoptosis, mitochondrial dysfunction, and neurodegeneration [24,25]. Artemisinin is a natural compound derived from *Artemisia annua*, which is widely available, inexpensive, and has low toxicity and rare side effects. It has been shown to have neuroprotective and anti-inflammatory effects by reducing ROS levels and restoring mitochondrial function [26,27,28]. The acetylation of histone is one of the most studied epigenetic markers in the neural retina. It plays an important role in retinal development, aging, and disease [29]. In this study, we investigated the effect of Artemisinin on histone acetylation in RPE cells exposed to H_2_O_2_ and its anti-apoptotic ability.

Our findings indicate that 500 μM H_2_O_2_ successfully induces oxidative stress in D407 cell cultures, an effect accompanied by a significant ROS generation at 24 h compared to the control group. It also caused cytotoxicity reflected by increased LDH release and thus induced cell viability decline. The marked increase in LDH release caused by H_2_O_2_ indicates that the cell plasma membrane has been damaged and that the cells may be undergoing apoptosis, necrosis, or other forms of cell death. In addition, the H_2_O_2_ caused a significant acute reduction in Acetyl-H4 (Lys 8) level that was more significant at 6 h than at 24 h. This observation is consistent with another study in which H_2_O_2_ temporarily reduced overall acetylation levels under chronic oxidative stress [30]. The increase in Acetyl-H4 (Lys 8) level at 24 h compared to that at 6 h may be due to a decrease in HDAC protein levels at 24 h, particularly HDAC4 (Supplementary Figure S1), which is sensitive to oxidative stress and prone to degradation in human lens epithelial cells, etc. [31,32,33]. At 6 h, H_2_O_2_ dose-dependently decreased Acetyl-H4 (Lys 8) and Acetyl-H3 (Lys 9) levels. Since histone acetylation is co-regulated by HATs and HDACs, these results suggest that H_2_O_2_ treatment may induce HDAC activity and repress HAT activity, leading to histone deacetylation and chromatin condensation. However, we observed that H_2_O_2_ did not induce the increased protein level of class I/IIα HDACs in D407 cell cultures. Whether H_2_O_2_ promotes the expression of class IIb, III, or IV HDACs or inhibits the expression of HATs is worthy of further investigation in the future.

Previous studies from our laboratory have shown that Artemisinin could attenuate the H_2_O_2_-induced increase in ROS levels, the decline in the mitochondrial membrane potential, and cell apoptosis in D407 cell cultures [14,15]. The current study confirms that pretreatment with Artemisinin blocked H_2_O_2_-induced D407 cell viability decline, as previously reported [14,15], and further expanded the protective mechanism of Artemisinin. This study showed that Artemisinin inhibited the decrease in Bcl-2 protein level caused by H_2_O_2_, suggesting that the effect of Artemisinin in protecting D407 cells from H_2_O_2_-induced apoptosis is partly dependent on the anti-apoptotic protein Bcl-2. Bcl-2 is a member of the Bcl-2 family of proteins that regulate the mitochondrial pathway of apoptosis by controlling the release of cytochrome c and other apoptogenic factors [23]. Most importantly, Artemisinin pretreatment also prevented the H_2_O_2_-induced reduction in Acetyl-H4 (Lys 8) level but not the Acetyl-H3 (Lys 9) level at both 6 h and 24 h in D407 cell cultures. Artemisinin pretreatment also increased the Acetyl-H4 (Lys 8) level and anti-apoptotic protein Bcl-2 expression level compared to the H_2_O_2_-treated group in another RPE cell line ARPE19 cells. These results indicate that Artemisinin protects RPE cells from H_2_O_2_-induced oxidative injury by modulating histone H4 (Lys 8) acetylation level, which may be involved in the regulation of mitochondria anti-apoptotic protein Bcl-2.

TSA is a potent and specific inhibitor of HDACs. It exerted cytoprotective effects in ARPE19 cells by improving antioxidant capacity and inhibiting inflammation [34], in retinal ganglion cells by upregulating Acetyl-H3/4 level, attenuating cell loss, and facilitating neuritogenic action [12,35], and also in rod and cone photoreceptor cells by reducing Poly (ADP-ribose) polymerase (PARP) activity and preventing degeneration [22,36,37], while it also protected retina from ischemic injury [38]. Our present findings showed that the acute increase in the acetylation level through inhibiting histone deacetylase by TSA could prevent H_2_O_2_-induced inhibition of the Acetyl-H4 (Lys 8) level and protect D407 cell cultures from H_2_O_2_-induced cell viability decrease and apoptosis, suggesting an apparent correlation between the increase in the Acetyl-H4 (Lys 8) level and anti-apoptotic effect. In the present study, TSA pretreatment also increased the Acetyl-H4 (Lys 8) level and anti-apoptotic protein Bcl-2 expression level compared to the H_2_O_2_-treated group in ARPE19 cells. The cytoprotective effects of Artemisinin and TSA were also identified in human primary RPE cells, suggesting a general effect. These results further indicate that the cytoprotective effect of Artemisinin toward H_2_O_2_-induced apoptosis in RPE cells is correlated to the increased Acetyl-H4 (Lys 8) level.

In conclusion, our findings support the mechanistic hypothesis that Artemisinin confers cytoprotection toward H_2_O_2_-induced apoptosis by inhibiting the H_2_O_2_-induced decrease in the acetylation level of histone H4 at Lys 8 in RPE cells. Our findings inspire some future directions and questions for further research. For example, what are the precise molecular mechanisms of how Artemisinin regulates histone acetylation and apoptosis in RPE cells? How does Artemisinin affect other histone modifications, such as methylation and phosphorylation, and their interactions with acetylation? How does Artemisinin affect the expression of histone acetyltransferases and deacetylases in RPE cells? Our findings also provide some recommendations and directions for future research on the role of histone acetylation in AMD pathogenesis and therapeutics. For the prospects of the study, our study has identified promising targets that can be exploited to develop novel therapeutic strategies for AMD. The hypothesis of using Artemisinin to modulate the balance between acetylation and deacetylation offers a potential avenue for therapeutic intervention. We believe that further exploration of this hypothesis could lead to the development of effective treatments for AMD. Further preclinical studies using animal models to validate our findings and assess the therapeutic potential of Artemisinin are needed. These studies will provide crucial insights into the translational potential of our work and inform the design of future clinical trials. There is also potential to combine epigenetic approaches with other therapeutic strategies, such as gene therapy or immunomodulation, to achieve synergistic effects in AMD treatments. Overall, further in vitro and in vivo investigation is deserved and may provide the basis for future clinical use of Artemisinin in the prevention and/or treatment of AMD.

## 4. Materials and Methods

### 4.1. Drugs, Regents, and Kits

MTT was purchased from Molecular Probes (Eugene, OR, USA). DMSO and H_2_O_2_ were obtained from Sigma-Aldrich (St. Louis, MO, USA). Artemisinin was purchased from Meilun Biotech Co. Ltd. (Dalian, China). TSA was received from Calbiochem (San Diego, CA, USA). Lactate dehydrogenase (LDH) Release Assay Kit and ROS Assay Kit were obtained from Beyotime (Shanghai, China). Annexin V—FITC/PI Kit was obtained from Sangon Biotech (Shanghai, China). The antibody name, clonality, source, dilution, and company are presented in Table 1.

### 4.2. Cell Culture

The D407 cell line was obtained from the cell bank at Sun Yat-sen University (Guangzhou, China). The ARPE19 cell line was bought from Shanghai Kanglang Biotechnology Co., Ltd. (Shanghai, China). The primary human RPE cells were obtained from the State Key Laboratory of Ophthalmology, Zhongshan Ophthalmic Center, Sun Yat-sen University (Guangzhou, China). D407 cells were cultured in Dulbecco’s modified Eagle’s medium (DMEM, GIBCO, Grand Island, NY, USA). ARPE19 cells and primary human RPE cells were cultured in DMEM/F12 (GIBCO), both DMEM and DMEM/F12 were supplemented with 10% fetal bovine serum (FBS, GIBCO), 100 units/mL penicillin, and 100 μg/mL streptomycin (GIBCO) at 37 °C in a humidified incubator with 5% CO_2_.

### 4.3. Cell Viability Assay

D407 cells (5 × 10^3^ cells/well) and primary human RPE cells (1 × 10^4^ cells/well) were applied and cultured in the 96-well plate. On the second day, the cell cultures were exposed to H_2_O_2_ after pretreatment with or without Artemisinin or TSA diluted in the FBS-free medium. After H_2_O_2_ treatment (500 μM) for 24 h, the cell cultures were incubated with 0.5 mg/mL MTT for 3 h. Thereafter, the formazan crystals formed and were dissolved with dimethyl sulfoxide (DMSO) (100 μL/well). The absorbance was detected at 570 nm by Victor X5 Microplate Reader (PerkinElmer, Waltham, MA, USA) to measure the cell viability. Results were normalized to the untreated control and expressed as a percent.

### 4.4. LDH Release Assay

D407 cells (5 × 10^3^ cells/well) were applied and cultured in the 96-well plate. On the second day, the cells were treated with H_2_O_2_ (500 μM) diluted in the FBS-free medium. After the treatment with H_2_O_2_ for 6, 12, or 24 h, the supernatant was transferred to the new 96-well plate and incubated with LDH detection working solution, protected from light, with slow shaking for 30 min at room temperature, according to the instruction manual (https://www.beyotime.com/product/C0017.htm, accessed on 30 March 2024). The absorbance was detected at 490 nm by Victor X5 Microplate Reader (PerkinElmer, Waltham, MA, USA). Results were normalized to the untreated control and expressed as a percent.

### 4.5. Flow Cytometry

D407 cells attached to the 6-well plate (1 × 10^5^ cells/well), and primary human RPE cells attached to the 12-well plate (5 × 10^5^ cells/well) were treated with 250 nM TSA for 2 h or 1 h, followed by incubation with 500 μM H_2_O_2_ for 24 h. Thereafter, the cultures were washed with PBS and digested with 0.25% trypsin. After centrifugation, the cells were resuspended in the binding buffer and stained with Annexin V-FITC and PI at room temperature, in the dark.

Cell apoptosis was detected by BD Accuri™ C6 Plus flow cytometer (BD Biosciences, San Diego, CA, USA), and analyzed by BD CSampler^TM^ Plus Software (version 1.0.23.1). The upper right quadrant represents late apoptotic cells and the lower right quadrant represents early apoptotic cells, and the apoptosis rate is the sum of the percentages of these two quadrants.

### 4.6. ROS Assay

D407 cells (5 × 10^3^ cells/well) were applied and cultured in the 96-well plate. On the second day, cells were treated with H_2_O_2_ (500 μM) diluted in the FBS-free medium for 6, 12, and 24 h. Thereafter, the cells were stained with the DCFH-DA (10 μM/L) probe at 37 °C in the cell culture incubator for 40 min. The green fluorescence was observed, and pictures were taken with EVOS™ M7000 microscope (Thermo Fisher Scientific, Waltham, MA, USA) (magnification ×20). The fluorescence values were measured with the Victor X5 Microplate Reader (PerkinElmer, Waltham, MA, USA) to quantify the ROS levels. Results were normalized to the untreated control and expressed as a percent.

### 4.7. Western Blotting

D407 cells attached to the 12-well plate (1.5~2.5 × 10^5^ cells/well) and ARPE19 cells attached to the 24-well plate (2.5 × 10^5^ cells/well) were exposed to H_2_O_2_ after pretreatment with or without Artemisinin or TSA diluted in the FBS-free medium. After 6 h or 24 h later, the cell cultures were harvested using cell lysis buffer, and the cell lysate was boiled at 100 °C for 10 min. The cellular protein was separated by gel electrophoresis using 4–20% SurePAGE™ precast gels (GenScript, Piscataway, NJ, USA), and then transferred to the PVDF membranes. The membranes were blocked with 2.5% (*w*/*v*) bovine serum albumin (BSA) at room temperature for 1 h with shaking, followed by incubation with diluted primary antibody at 4 °C overnight, with agitation. After removing the primary antibody, membranes were washed with TBST three times for 30 min at room temperature on the orbital shaker (Bluepard, Shanghai, China) with the speed of 100 r/min, then incubated with diluted secondary antibody for 1.5 h at room temperature with shaking. After removing the secondary antibody, the membranes were washed with TBST as above, and exposed to BIO-RAD ChemiDoc Touch Imaging System (Bio-Rad, Hercules, CA, USA) after adding Clarity Western ECL Substrate (Bio-Rad, Hercules, CA, USA). The protein bands were quantified by Image J (version 1.51d).

### 4.8. Data Analysis

Each experiment was repeated at least three times. The data are presented as mean ± SEM. The statistical comparison was performed using GraphPad Prism 5.0 statistical software (San Diego, CA, USA). One-way ANOVA followed by Tukey’s multiple comparisons was used to determine the significance between each group. * indicates *p* < 0.05, ** indicates *p* < 0.01, *** indicates *p* < 0.001.

## Figures and Tables

**Table 1:** List of antibodies used for western blotting in this study.

Antibody Name	Clonality	Source	Dilution	Company (Catalog)
GAPDH	Monoclonal	Mouse	1:1000	SAB (40,493)
Acetyl-Histone 4 (Lys 8)	Polyclonal	Rabbit	1:1000	CST (2594)
Acetyl-Histone 3 (Lys 9)	Polyclonal	Rabbit	1:1000	CST (9671)
Bcl-2	Monoclonal	Rabbit	1:1000	CST (3498)
Bax	Polyclonal	Rabbit	1:1000	CST (2772)
HDAC1	Polyclonal	Rabbit	1:1000	CST (2062)
HDAC2	Polyclonal	Rabbit	1:1000	CST (2540)
HDAC3	Polyclonal	Rabbit	1:1000	CST (2632)
HDAC4	Polyclonal	Rabbit	1:1000	CST (2072)
HDAC5	Polyclonal	Rabbit	1:1000	CST (2082)
HDAC7	Polyclonal	Rabbit	1:1000	CST (2882)
Anti-mouse IgG HRP-conjugated, secondary antibody	Polyclonal	Horse	1:2000	CST (7076)
Anti-rabbit IgG HRP-conjugated, secondary antibody	Polyclonal	Goat	1:2000	SAB (L3012)

GAPDH: glyceraldehyde-3-phosphate dehydrogenase; Bcl-2: B-cell lymphoma 2; Bax: Bcl-2 Associated X-protein; HDAC: histone deacetylase; HRP: horseradish peroxidase; CST: Cell Signaling Technology (Danvers, MA, USA); SAB: Signalway antibody (Greenbelt, MD, USA).

## Data Availability

Data are contained within the article and Supplementary Materials.

## Supplementary Materials

The following supporting information can be downloaded at: https://www.mdpi.com/article/10.3390/molecules29081789/s1, Figure S1. Representative western blotting image of HDAC4 protein level. The D407 cells were treated with different concentrations of H_2_O_2_ (125, 250, or 500 µM) for 24 h. HDAC4 and GAPDH protein levels were detected by western blotting.
