# Supplementary material for: Artemisinin Confers Cytoprotection toward Hydrogen Peroxide-Induced Cell Apoptosis in Retinal Pigment Epithelial Cells in Correlation with the Increased Acetylation of Histone H4 at Lysine 8
Source: Molecules. 2024 Apr 15;29(8):1789. doi: 10.3390/molecules29081789 (PMC11051841; doi:10.3390/molecules29081789)
Supplement: Supplementary file 1 [file molecules-29-01789-s001.zip › molecules-2911508-supplementary.pdf]

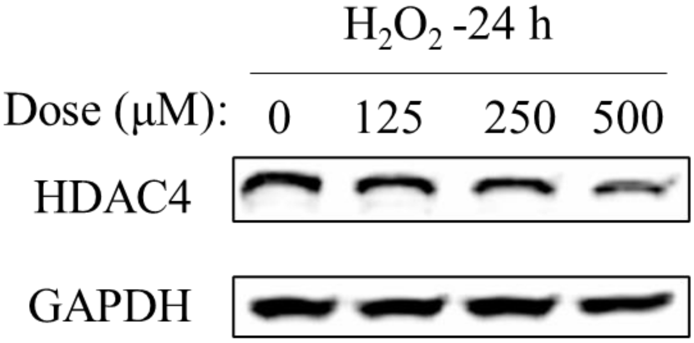

Figure S1. Representative western blotting image of HDAC4 protein level. The D407 cells were treated with different concentrations of H_2O_2 (125, 250, or 500 μM) for 24 h. HDAC4 and GAPDH protein levels were detected by western blotting.
